# Designed Coiled-Coil Peptides Inhibit the Type Three Secretion System of Enteropathogenic *Escherichia coli*

**DOI:** 10.1371/journal.pone.0009046

**Published:** 2010-02-04

**Authors:** Mariano Larzábal, Elsa C. Mercado, Daniel A. Vilte, Hector Salazar-González, Angel Cataldi, Fernando Navarro-Garcia

**Affiliations:** 1 Instituto de Biotecnología, Instituto Nacional de Tecnología Agropecuaria (INTA), Castelar, Buenos Aires, Argentina; 2 Instituto de Patobiología, Instituto Nacional de Tecnología Agropecuaria (INTA), Castelar, Buenos Aires, Argentina; 3 Departamento de Biología Celular, Centro de Investigación y Estudios Avanzados (Cinvestav), México DF, Mexico; Charité-Universitätsmedizin Berlin, Germany

## Abstract

**Background:**

Enteropathogenic *E. coli* (EPEC) and enterohemorrhagic *E. coli* (EHEC) are two categories of *E. coli* strains associated with human disease. A major virulence factor of both pathotypes is the expression of a type three secretion system (TTSS), responsible for their ability to adhere to gut mucosa causing a characteristic attaching and effacing lesion (A/E). The TTSS translocates effector proteins directly into the host cell that subvert mammalian cell biochemistry.

**Methods/Principal Findings:**

We examined synthetic peptides designed to inhibit the TTSS. CoilA and CoilB peptides, both representing coiled-coil regions of the translocator protein EspA, and CoilD peptide, corresponding to a coiled–coil region of the needle protein EscF, were effective in inhibiting the TTSS dependent hemolysis of red blood cells by the EPEC E2348/69 strain. CoilA and CoilB peptides also reduced the formation of actin pedestals by the same strain in HEp-2 cells and impaired the TTSS-mediated protein translocation into the epithelial cell. Interestingly, CoilA and CoilB were able to block EspA assembly, destabilizing the TTSS and thereby Tir translocation. This blockage of EspA polymerization by CoilA or CoilB peptides, also inhibited the correct delivery of EspB and EspD as detected by immunoblotting. Interestingly, electron microscopy of bacteria incubated with the CoilA peptide showed a reduction of the length of EspA filaments.

**Conclusions:**

Our data indicate that coiled-coil peptides can prevent the assembly and thus the functionality of the TTSS apparatus and suggest that these peptides could provide an attractive tool to block EPEC and EHEC pathogenesis.

## Introduction

Enteropathogenic *E. coli* (EPEC) and enterohemorrhagic *E. coli* (EHEC) are associated with diarrhea in humans. EPEC is a major cause of infantile diarrhea in developing countries [1] and EHEC is responsible for disease whose clinical spectrum includes diarrhea, hemorrhagic colitis and hemolytic uremic syndrome (HUS), the leading cause of renal failure in children in Argentina and several other countries [2], [3], [4]. Shiga toxin expression from integrated bacteriophages in EHEC strains [2], [5] is considered responsible for both hemorrhagic colitis and HUS. The main reservoir of EHEC is healthy cattle although a limited number of serogroups have been associated with diarrhea in young calves [5], [6], [7], [8].

Both categories of *E. coli*, EPEC and EHEC, are known to carry a locus of enterocyte effacement pathogenicity island (LEE) [9], [10], [11]. The LEE encodes a type III secretion system (TTSS) [12] that injects effector proteins into enterocytes, some of which alter signaling pathways. This process results in the formation of “attaching and effacing” (A/E) lesions on intestinal epithelia, which are characterized by the intimate adhesion of bacteria to actin-rich pedestals and a localized destruction of microvilli. The TTSS is a complex structure of more than 20 proteins forming a ‘needle and syringe’ apparatus that allows effector proteins to be injected directly into the host cell [13], [14], [15].

The LEE has been completely sequenced and contains five major operons: *LEE1*, *LEE2*, *LEE3*, *LEE4* and *LEE5*. EspA [16], EspB and EspD [17] are some of the proteins encoded by *LEE4* that make up the translocon portion of the TTSS [11], [18]. EspA makes hollow, filamentous appendages surrounding the bacteria, which are present in a transient manner [18]. These structures form a translocation tube that acts as a channel to deliver proteins from the bacteria into the intestinal cell. EspB and EspD are involved in pore formation on the membranes of the infected cells [19] and are translocated to both the membrane and the cytoplasm [20]. Complexes formed by EspA, EspB and EspD proteins may participate in the initial step of bacterial adherence [21]. The proximal end of the EspA filament rests in the EscF needle near the basal body of the TTSS [22]. EscF is also encoded in *LEE4*. *LEE5* encodes the bacterial outer membrane protein intimin, responsible for the intimate attachment of the bacteria to host enterocytes, and its own receptor Tir, which is translocated through the TTSS into the host cell surface [23].

Coiled-coil regions are involved in protein-protein interaction, especially in the formation of multimeric complexes and molecular recognition [24], [25]. Coiled-coil regions comprise alpha-helices interlaced around each other in a highly organized manner. A regular sequence pattern known as the heptad repeat, a seven-residue pattern denoted *abcdefg* in which the *a* and *d* residues are hydrophobic, is the basis of coiled coils. Coiled-coil sequences have an important role in the formation of TTSS structures [26], [27]. The carboxy terminus of EspA comprises an alpha-helical region which demonstrates heptad periodicity. Site-directed mutagenesis of EspA heptad residues has generated EPEC mutants defective in EspA filament assembly, indicating that coiled-coil interactions are essential in the assembly of EspA filament associated with the TTSS [27]. In this study, we designed synthetic peptides corresponding to coiled-coil domains of EspA, EscF and to the intimin interacting region of Tir, which were tested for inhibiting the action of TTSS.

## Results

### Designed Peptides Inhibit TTSS-Mediated RBC Lysis

Many TTSS protein are predicted to share a common coiled-coil structural feature. In fact, the coiled-coil domain of EspA is required for assembly of the EspA filament-associated type III secretion translocon [25]. Therefore, peptides were designed to target coiled-coil domains in EspA and EscF. The coiled-coil region of EspA contains 30 aa. To better discriminate domains inside this region two 15-aa peptides were designed. CoilA and CoilB peptides contain 15 aa corresponding to coiled-coil domains at the C-terminal end of EspA [26], [15]. CoilC and CoilD represent N-terminal coiled-coil domains of EscF [15] and PepTir peptide represents the interaction region between Tir and its ligand, intimin [28] (Table 1).

**Table 1:** Characteristics of designed peptides.

Name	Target	Sequence
CoilA	C-terminal Sequence of EspA	[**L**TTTVNN][**S**QLEIQQ]M
CoilB	C-terminal Sequence of EspA	[**M**SNTLNL][**L**TSARSD]M
CoilC	N-terminal Sequence of EscF	LSDSVPELLNSTDLV
CoilD	N-terminal Sequence of EscF	VNDPEKMLELQFAVQ
PepTIR	Tir	KVNIDENGNAI

Heptads is shown between square brackets and the *a* position of heptads is in bold.

**Figure 1:**
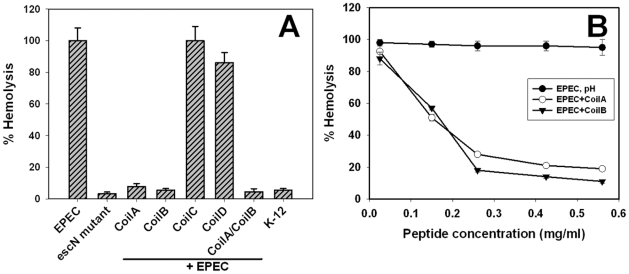
Coiled-coil peptides effectively inhibit RBC hemolysis promoted by EPEC. (A) Hemolysis of sheep RBC observed with EPEC bacteria preincubated with coiled-coil peptides. Results are presented as percentage relative to hemolysis **(±** standard deviation) observed upon incubation of RBC with EPEC alone. K12 means the hemolysis caused by the *E. coli* non-pathogenic strain. (B) Doses-dependent effect of CoilA and CoilB peptides on the hemolysis of RBC. The peptides were applied to the RBC-E2348/69 system to various concentrations and a dose-dependent response was observed. pH represent a mock control to rule out the biological influence of alkaline pH used to dissolve peptides. Each assay was done by triplicate.

Twelve-well plates containing peptides and bacteria were incubated for 1 h to allow their interaction. A 5% suspension of RBC was then added, plates were incubated for another 3 h, and supernatants were monitored for released hemoglobin. The EPEC E2348/69 strain produced hemolysis (referenced as 100%), whereas EPEC Δ*escN*, a mutant that does not synthesize the TTSS, produced only 3% lysis, which is usual for a non-pathogenic strain (*E. coli* K-12), thus indicating that the RBC lysis was due to the TTSS (Fig. 1A). CoilA and CoilB peptides inhibited around 95% of the TTSS-mediated RBC lysis caused by the E2348/69 strain; this inhibitory effect was not synergistic since preincubation of EPEC with both peptides also caused a hemolysis inhibition of 95% (Fig. 1A). Other coiled-coil structures, such as CoilD, reduced the hemolysis by only 25%. Interestingly, in all cases, the inhibitory effect was observed only when the bacteria were preincubated with the corresponding peptide, since peptides did not reduce the hemolysis when added to the bacteria at the same time as RBC. On the other hand, CoilC (Fig. 1) and PepTir (data not shown) did not show inhibitory activity. Inhibition of hemolysis caused by EPEC due to CoilA and CoilB peptides was dose-dependent since a reduction of CoilA peptide concentration to 0.15 mg/ml caused a 50% of hemolysis. We chose to use peptides at 0.56 mg/ml in spite of the fact that a concentration of 0.36 mg/ml is enough to cause 90% of inhibition (Fig. 1B).

### Designed Peptides Inhibit the Formation of A/E Lesions

**Figure 2:**
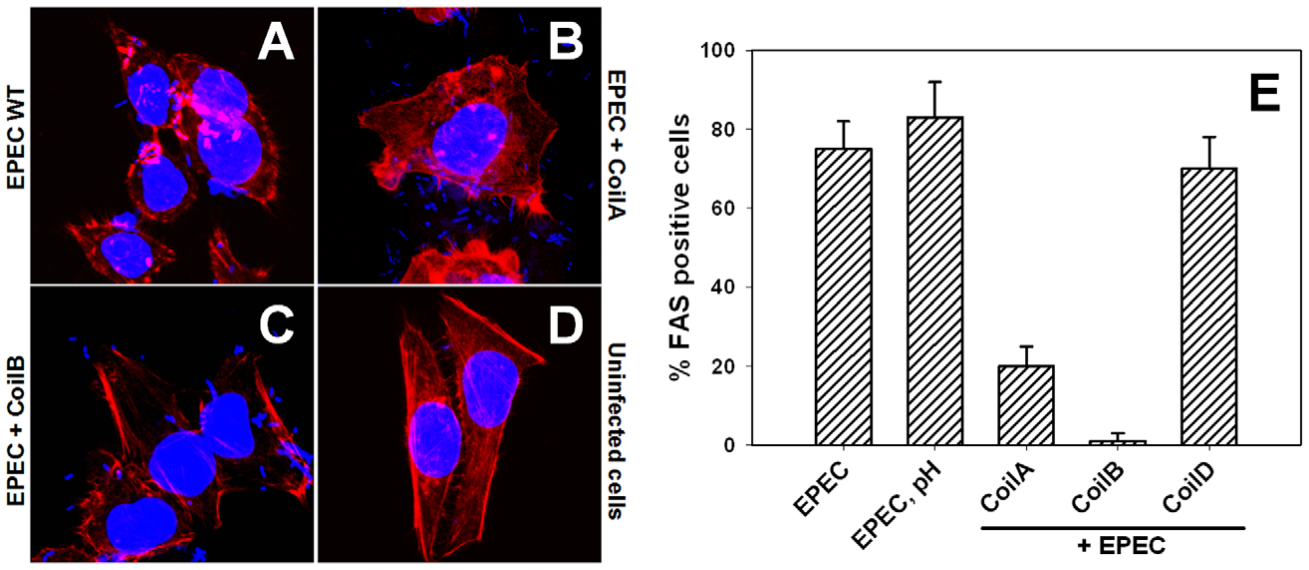
Coiled-coil peptides inhibit A/E lesion formation caused by EPEC. HEp-2 cells were incubated with EPEC E2348/69 either with or without coiled-coil peptides. Fluorescent actin staining polymerization was revealed using rhodamine phalloidin (red) and TO-PRO-3 to stain bacterial and eukaryotic DNA (blue) and imaged by confocal microscopy. Incubation of HEp-2 cells with the wild type EPEC E2348/69 strain without peptides (EPEC WT); preincubated with CoilA (EPEC+CoilA), or CoilB (EPEC+CoilB) peptide and uninfected cells. The corresponding percentage of HEp-2 cells carrying pedestals, obtained by examining 100 cells, is shown in panel E. EPEC pH represents a mock control to rule out the biological influence of alkaline pH used to dissolve peptides; PBS at alkaline pH was used in volumes identical to those in which peptides were added to HEp-2 cells.

Since CoilA, CoilB and CoilD were able to inhibit the hemolysis caused by EPEC, which could involve translocator proteins at cell membrane level, we decided to explore whether peptides were also able to block effector proteins delivery and thus prevent the formation of A/E lesions in epithelial cells. Thus, peptides interfering in actin polymerization beneath E2348/69 strain adhering to HEp-2 cells were visualized by FAS (fluorescent actin staining). As expected, EPEC bacteria were able to induce actin polymerization for pedestal formation in almost all the cells (Fig. 2A), the A/E lesions were not seen in uninfected cells (Fig. 2B). CoilA or CoilB preincubation with EPEC caused a decrease in pedestal formation in epithelial cells, showing different effectiveness (Fig. 2C and Fig. 2D). A morphometric study of different fields by confocal microscopy showed that CoilB peptide strongly inhibited the A/E lesion formation by EPEC, reducing the number of HEp-2 cells showing pedestals to 2% (P<0.01), as compared with untreated cells (EPEC without peptides), where 80% of cells had pedestals (Fig. 2E). On the other hand, CoilA reduced pedestal-forming cells to 20% (P<0.01), whereas CoilD was unable to inhibit pedestal formation in HEp-2 cells (Fig. 2E) and the percentage of Fas-positive cells was similar to those cells treated with EPEC at the pH used to dissolve the peptides.

### Designed Peptides Interfere with EspA Polymerization and Tir Translocation

**Figure 3:**
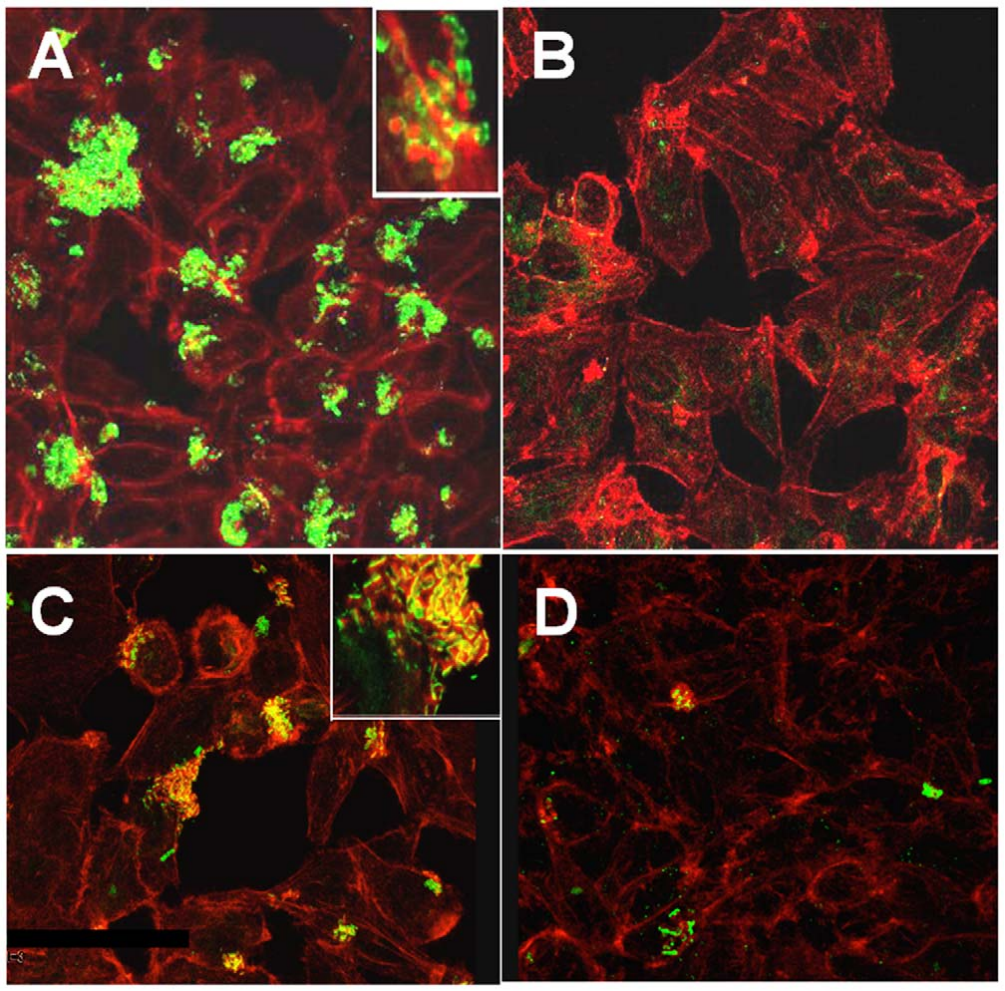
Coiled-coil peptides inhibit EspA polymerization and Tir translocation. Confocal microscopy images (1000X magnification) showing fluorescence on HEp-2 cells infected with EPEC E2348/69 and either with or without coiled-coil peptides. Incubation of HEp-2 cells with (A) the wild type EPEC E2348/69 strain, without peptides. Actin was revealed by FAS using rhodamine-phalloidin (red) and EspA polymers by anti EspA sera (green); (B) the wild type EPEC E2348/69 strain preincubated with CoilA peptide, staining as in A; (C) the wild type EPEC E2348/69 strain, without peptides. Actin was revealed by FAS using rhodamine-phalloidin (red) and Tir by anti Tir sera (green). Colocalization of F-actin and Tir appears as yellow; (D), the wild type EPEC E2348/69 strain preincubated with CoilA peptide, staining as in C. The insets (magnification 5000 x) show additional details.

Since both peptides, CoilA and CoilB, are directed against EspA and inhibited pedestal formation, we determined their efficiency to block EspA polymerization and thereby Tir translocation. For that, HEp-2 cells were treated with EPEC preincubated either with or without CoilA or CoilB, and EspA and Tir were then visualized using specific antibodies and confocal microscopy. A drastic decrease in the secreted EspA (Fig. 3B) and translocated Tir (Fig. 3D) was observed in bacteria preincubated with CoilA. Similar results were obtained when CoilB was used (data not shown). In HEp-2 cells monolayers treated with EPEC without peptides, as expected, expression of EspA on the infecting bacteria was detected (Fig. 3A) and colocalization of Tir and F-actin was observed (Fig. 3C). A HEp-2 detaching activity by EPEC was observed in control cultures. In contrast, this cytopathic effect was absent when bacteria were preincubated with CoilA or CoilB peptides (data not shown). Furthermore, we observed that peptides did not inhibit the growth of E2348/69 in LB media (data not shown). We also noticed that coiled-coil peptides decreased the EPEC adherence to HEp-2 cells by detecting more non-adherent bacteria in the supernatant of HEp-2 cells treated with EPEC plus CoilA (7 fold) or CoilB (40 fold) than in cells treated with EPEC but without peptides (data not shown).

**Figure 4:**
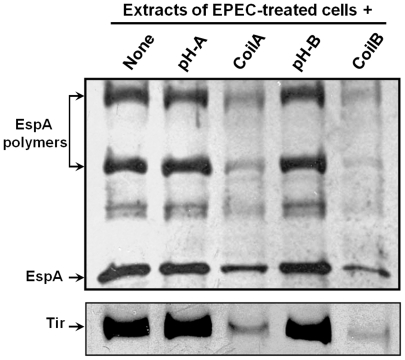
CoilA and CoilB peptides block EspA polymerization and Tir translocation. Cell monolayers were infected with bacteria pretreated with peptides, washed, and lysed for Western blot detection of EspA and Tir proteins with specific rabbit antisera. pH represents a mock control to rule out the biological influence of alkaline pH used to dissolve peptides; PBS at alkaline pH was used in a volume identical to those in which peptides were added to HEp-2 cells.

To further determine the effect of CoilA and CoilB on EspA polymerization and Tir translocation were also examined by Western blotting. For that, HEp-2 cells were treated for 2 h with either EPEC alone or preincubated with each of the peptides and exhaustively washed. Cell extracts were prepared and the contents of EspA and Tir (Fig. 4) analyzed by Western blot using specific antibodies. CoilA and CoilB drastically decreased the amount of EspA associated to the cells and attached bacteria (Fig. 4), but, more importantly, the amount of EspA species detected as EspA polymers (see arrowhead in Fig. 4). These effects correlated with a significant decrease of Tir in the cells (Fig. 4) from the same preparations. The effective amount of EspA and Tir associated per cell may be even higher in non-treated (without peptides) cell cultures because it could be underestimated as some HEp-2 cells are detached from the surface upon infection. The pH buffering solution used to dilute CoilA and CoilB peptides had no effect and the results were similar to the positive control (Fig. 4).

**Figure 5:**
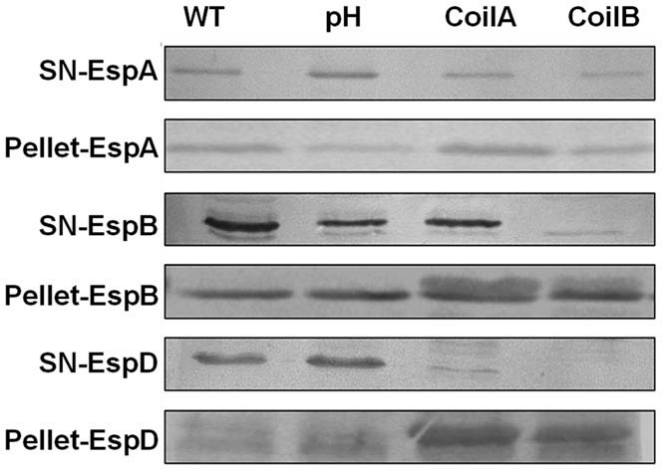
Coiled-coil peptides affect the secretion of EspB and EspD but not EspA secretion. EPEC E2348/69 bacterial cultures were incubated in the presence of CoilA or CoilB peptides and concentrated supernatants and bacterial extracts were analyzed by Western blotting using specific antisera directed to EspA, EspB, EspD or Tir. The same amount of cell monolayer was loaded in each lane. pH represents a mock control to rule out the biological influence of alkaline pH used to dissolve peptides. PBS at alkaline pH was used in volumes identical to those in which peptides were added to cultures.

To determine whether CoilA and CoilB peptides have an effect on protein secretion by the TTSS *in vitro*, EPEC bacteria were incubated for 4 h either with or without peptides, in the same conditions as for RBC lysis and pedestal formation assays, and EspA, EspB, EspD and Tir proteins were sought in supernatants and bacterial lysates. Bacterial lysates or supernatants of E2348/69 cultures incubated with CoilA or CoilB peptides showed similar amounts of cell-associated and secreted EspA, comparable with the amounts showed by bacteria incubated without peptides, as determined by Western blotting (Fig. 5). In contrast, preincubation of bacteria with CoilB blocked the secretion of EspB to the culture supernatant (Fig. 5). This effect was not clearly observed with CoilA. On the other hand, secretion of EspD was affected by both peptides, CoilA and CoilB, and the protein was detected in the pellet of lysed bacteria. CoilA or CoilB lightly decreased the secretion of Tir. The fact, that CoilA and CoilB did not affect the intrabacterial content of Esp proteins indicates that gene expression of these proteins is not distorted by coiled-coil peptides. As expected, the Δ*escN* strain a mutant that does not synthesize the TTSS showed no EspA in culture supernatants, thus confirming the absence of bacterial lysis (data not shown).

### CoilA Peptide Blocks Formation of EspA Filaments on the Bacteria

**Figure 6:**
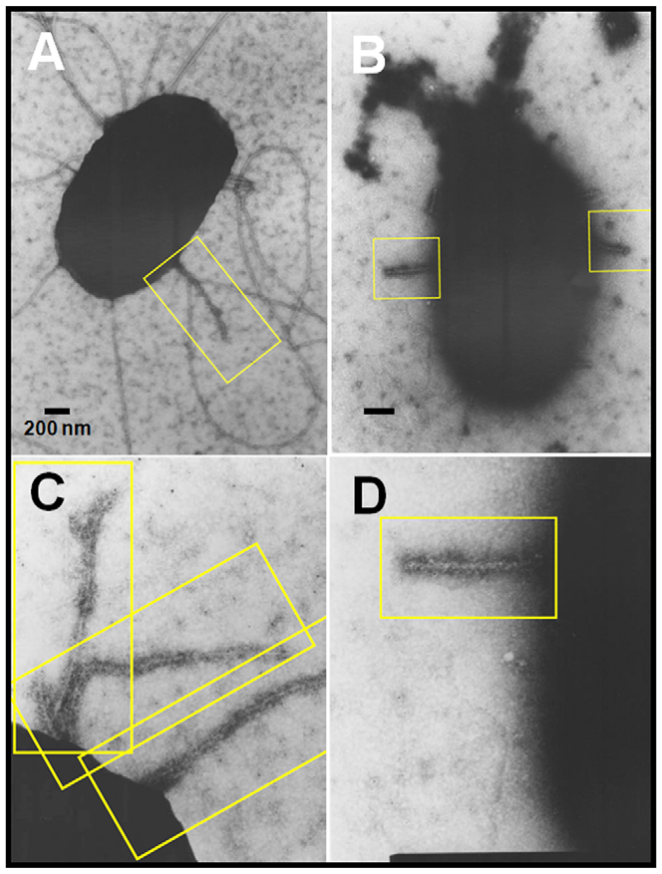
CoilA peptide blocks the formation of EspA filaments. Immunogold EM of EspA filaments on EPEC cultures incubated either with or without CoilA peptide. After incubation of bacterial samples with specific rabbit anti-EspA serum, the samples were observed by TEM. EPEC alone, without peptides (A, C) and EPEC incubated with CoilA (B, D). Bars, 200 nm (A, B), or 100 nm (C, D). Yellow box frames show EspA filaments.

Since coil peptides are able to inhibit the TTSS by blocking EspA polymerization, we expected a decrement in EspA filaments on the bacterial cell surface upon incubation with coil peptides. To assess this hypothesis, EPEC was subjected to immunogold staining after *in vitro* induction of TTSS-synthesis in either the presence or absence of CoilA peptide, since Coil B has a tendency to precipitate forming clumps that interfere with the quality of electron micrographs. Indeed, transmission electron microscopy analysis showed EspA filaments with an average length of 700±150 nm displayed on EPEC bacteria, which were detected by anti-EspA and immunogold. Interestingly, bacteria preincubated with CoilA showed shorter EspA filaments, which were reduced to a length of about 250±50 nm (Fig. 6). This result correlates with the observed reduction in EspA protein as monomer and polymer species in lysed cells infected with EPEC in the presence of either CoilA or CoilB peptides (see Fig. 4).

## Discussion

Various pathogenic bacteria use related virulence systems, contradicting the long-held paradigm that each bacterium has a unique mode of action. These findings have implications for development of novel antibacterial agents as well as research aimed at understanding bacterial virulence. The type III secretion systems are virulence mechanisms developed by many Gram-negative pathogenic bacteria to introduce effector proteins into the cytoplasm of eukaryotic cells. In all relevant model systems tested thus far, mutating or deleting a part of the TTSS significantly decreases the virulence of the affected pathogen. Presumably, chemical compounds that specifically inhibit the type III system may prevent disease by impairing essential virulence properties within this system. Thus, this strategy to target virulence is attractive [29], [30], [31]. A general method used today within the pharmaceutical industry and academia to find starting points for drug development is to screen large collections of compounds in various assays [32]. These large-scale screens are generally termed high-throughput screening [33]. Hits from the screening are further evaluated to verify that the identified response is not due to side effects (false positives). With this complicated method, it is possible to identify small-molecule virulence inhibitors in the absence of target structural information. However, since the TTSS was first identified, important advances have been achieved in understanding the genetics, structure and function of the TTSS [13]. Thereby, in this work we designed peptides with sequences derived from coiled-coil sequences from main components of the TTSS from EPEC and EHEC. We found that CoilA and CoilB were able to block EspA assembly, thus destabilizing the TTSS and Tir translocation, as well as inhibiting EspB and EspD secretion.

A class of small-molecule inhibitors specifically targeting the TTSS has been identified both in *Yersinia* spp. after screening a library of 9,400 compounds [34], [35] and in EPEC after screening 20,000 compounds [36]. In addition, the same or similar compounds have also been shown to inhibit the TTSS in *Chlamydia trachomatis* [37] and Salmonella TTSS-1 [38]. However, this compound was initially identified in a phenotype-based reporter-gene assay and, although additional studies have indicated TTSS specificity, the target protein is still unknown [39]. Here we specifically designed 15-aa peptides based on the coiled-coil structure of components of the TTSS of EPEC and EHEC bacteria. CoilB, which was designed to block the last 15 aa of the coiled-coil structure in the C-terminus of EspA, was the most efficient peptide in inhibiting EspA polymerization, while CoilA, which was designed to block the first 15 aa of the same coiled-coil structure, was less efficient than CoilB. Interestingly, CoilC and CoilD, which were designed to block the coiled-coil structure of EscF, were unable to block the A/E lesion, although CoilD showed a small effect of blocking the hemolysis by EPEC. At present, it is not clear whether both peptides are effective in preventing the coiled-coil association of EscF with itself and/or with EspA or whether that association is stronger than that of EspA multimerization. PepTir was ineffective in the hemolytic assay, which is in agreement with previous reports indicating that Tir is not necessary for RBC lysis [44]. The effect of PepTir should be tested in the future in FAS assays. These data suggest that the effects of the coiled-coil peptides are related to their availability during TTSS formation (i.e. during EspA polymerization instead of interfering in EscF and EspA interaction), as indicated by the fact that peptides are effective only when added to bacteria that are growing TSSS and not once it is already formed. It has been previously demonstrated that non-conservative amino acid substitution of specific EspA heptad residues in the coil structure generates EPEC mutants defective in filament assembly but which retain the ability to induce A/E lesions; an additional mutation can totally abolish EspA filament assembly and A/E lesion formation [26]. In the present work, we showed that CoilA and CoilB interfere with EspA filament as detected by confocal and electron microscopy and biochemically; this effect correlates with the inhibition of Tir translocation and A/E lesion.

Interestingly, CoilA and CoilB interfering with the EspA:EspA interaction during the filament formation also blocks the bacteria:host cells interaction, since in EPEC and EHEC the type III secreted protein EspA is assembled into a filamentous organelle that attaches the bacterium to the plasma membrane of the host cell. Formation of EspA filaments is dependent on the expression of another type III secreted protein, EspD, which is involved in the formation of the translocation pore in the host cell membrane. Although EspD does not appear to be a structural component of the EspA filament, an EspD EPEC mutant secretes only low levels of EspA and produces barely detectable EspA filaments [18]. EspD is translocated into the host cell membrane and is required for cell attachment [40] and EPEC-induced hemolysis [44]. Additionally, the carboxy terminus of EspD is predicted to adopt a coiled-coil conformation with 99% probability. In fact, there is an EspD-EspD protein interaction and a radical mutation in the C-terminus coiled-coil domain of EspD affects EPEC induced A/E lesion formation, EspA filament-mediated cell attachment, and EPEC induced hemolysis without affecting EspA filament biosynthesis [27]. Here, we also found a close relation between EspA and EspD, since CoilA or CoilB were able to block EspA filament formation as well as EspD secretion to the spent media. On the other hand, EspB mutants are unable to translocate Tir [23], thus suggesting that functional EspB is also required for protein translocation. EspB can bind and be copurified with EspA [41]. However, formation of EspA filaments and binding of EspA filaments to the target host cell can occur even in the absence of EspB [41], thus suggesting that EspB modulates EspA filament activity and signals the transition from an adhesive to a translocation function. Indeed, we found that even though CoilA or CoilB inhibit A/E lesion formation, only CoilB is able to inhibit EspB secretion to the spent media; perhaps this effect allows CoilB to inhibit EPEC infection more efficiently.

CoilA and CoilB peptides seem to have little or no effect in the *in vitro* secretion of EspA and Tir (Fig. 5). Particularly interesting is the case of Tir, because upon coiled-coil incubation, this effector protein was barely detected on HEp-2 cells by microscopy (Fig. 3D) or associated to HEp-2 cell by Western blot (Fig. 4). Taken these results together, it appears as if the translocation, but not the secretion of Tir is affected by coiled-coil peptides. These observations are congruent with a defect in the injectosome formation. Further research will clarify this point as well as whether, upon incubation with coiled coil peptides, tips of filament are capped with EspB and/or EspD. As EspD is not secreted in the presence of CoilA or CoilB peptides, EspD is probably not present in the tip of filaments of bacteria treated with coiled-coil peptides.

In conclusion, the design of molecules to impair intimate attachment of attaching and effacing pathogens to the intestinal mucosa will probably have an important effect on dangerous microorganisms of public health importance, and there is a possibility that virulence factor inhibitors will be effective for a long time before resistance becomes an issue. Research on finding new inhibitors for novel virulence targets can potentially be used therapeutically, or preventive as in this case, as well as be a tool for elucidating the intricate regulatory systems that modulate EPEC and EHEC virulence or any other bacterial member harboring a TTSS. Coiled-coil peptides are small molecules easier to synthesize than complex organic molecules and might be part of a nutraceutical formulation against diarrheogenic pathogens.

## Materials and Methods

### Ethics Statement

All research involving animals were conducted according to relevant national and international guidelines and approved by the Institutional Animal Care and Use Committee del Centro de Investigación en Ciencias Veterinarias y Agronómicas del INTA (CICUAE INTA-CICVyA), Argentina.

### Peptides, Bacterial Strains and Growth Media

CoilA, CoilB, CoilC, CoilD and PepTir peptides were synthesized commercially (Genbiotech, Buenos Aires). Peptides were solubilized with PBS (pH 10.0). CoilB was not completely soluble. Due to the alkalinity of the peptide solution, in hemolysis and FAS experiments, a similar amount of PBS pH 10.0 was used as a mock control to rule out the influence of pH in the effects observed. EPEC E2348/69 [42] was kindly provided by Marta Rivas, ANLIS-Instituto Nacional de Microbiología Dr. Carlos G. Malbrán, Buenos Aires. The *E. coli* EPEC E2348/69 Δ*escN* strain [43], a mutant that does not synthesize the TTSS was used as a negative control in the hemolysis and FAS assays. Bacteria were grown in Luria Bertani broth (LB) or Dulbecco's modified Eagle medium (DMEM) lacking phenol red (Gibco-BRL), without antibiotics and bovine fetal serum at 37°C without shaking.

### Red Blood Cell Lysis Assay

The possible inhibitory effect of peptides on the hemolytic activity exhibited by TTSS-encoding *E*. *coli* strains [44] was evaluated. The EPEC E2348/69 strain was grown in LB broth overnight at 37°C without shaking, OD_600_ was taken to measure the number of bacteria, and then diluted 1∶100 into Dulbecco's modified Eagle medium (DMEM) lacking phenol red (Gibco-BRL) with 0.56 mg/ml of peptides into 12-well plates. The plates were incubated for 1 h under a 5% CO_2_ atmosphere to allow the interaction between peptides and bacteria. In turn, red blood cells (RBCs) were separated by centrifugation from fresh defibrinated sheep blood, which was obtained by jugular vein punction, washed three times with 10 mM PBS (pH 7.4) and resuspended at 5% in PBS. Then, 2 ml of the 5% suspension of RBC in PBS was added to the cultures in the plates and incubated for 3 h at 37°C under a 5% CO_2_ atmosphere. The suspension was removed from the plates and centrifuged at 12,000×*g* for 1 min. Supernatants were monitored for the presence of released hemoglobin by measuring the OD at 543 nm.

### Immunofluorescence Staining

HEp-2 cells (5×10^4^) were seeded in eight well chamber slides (NUNC, Lab-Tek, USA) and incubated at 37°C under a 5% CO_2_ atmosphere up to 70–90% confluence. Overnight LB culture of the EPEC E2348/69 strain was diluted 1∶20 into 3 ml of DMEM and cultured for 1 h. CoilA, CoilB or CoilD peptides were then added to the culture at a concentration of 0.56 mg/ml and allowed to interact with the bacteria for 1 h at 37°C under a 5% CO2 atmosphere. Next, the bacteria concentration was adjusted measuring the OD_600_ to inoculate HEp-2 cells at a multiplicity of infection of 7 and 400 µl was added to the washed cells. After 2 h of interaction at 37°C in 5% CO2, cells were stained as described by Knutton et al. [45], with minor modifications. Briefly, the monolayers were gently washed three times with PBS, to eliminate the non-adherent bacteria, fixed in 4% paraformaldehyde in PBS for 20 min and permeabilized for 5 min in 0.15% Triton X- 100 in PBS. After three washes with PBS, the cells were stained by fluorescent actin staining (FAS) with tetramethyl rhodamine iso-thiocyanate (TRITC)-labeled phalloidin (Molecular Probes Europe, Leiden, The Netherlands) to stain actin. TO PRO-3 staining was used to detect eukaryotic nuclei and bacterial DNA (Invitrogen). In another similar experiments, EspA and Tir proteins were stained using rabbit specific antibodies obtained elsewhere [46] and fluorescein isothiocyanate (FITC)-conjugated goat anti-rabbit IgG (Zymed) as secondary antibody. The cells were observed under fluorescence microscopy with a confocal laser microscope (Leica TC SP2).

### Immunoblotting

In order to determine the effect of syntethic peptides in the production and excretion of TTSS-proteins by EPEC, cellular and supernatants culture extracts of peptide-treated bacteria were subjected to western blot analysis with specific antisera. The E2348/69 strain grown overnight at 37°C in LB was diluted 1∶100 into 2 ml of DMEM containing CoilA or CoilB peptides at a concentration of 0.56 mg/ml, respectively, and cultured for another 4 h in the presence of 5% CO_2_. After collecting by centrifugation, bacteria were suspended in 2X SDS sample buffer and boiled for 5 min. The proteins present in culture supernatants were precipitated by the addition of trichloroacetic acid at 20% (wt/vol) and incubated overnight at 4°C. The protein precipitates were then collected by centrifugation at 20,000×g for 25 min, washed twice with 200 µl of ice-cold acetone and resuspended in 20 µl Tris-HCl 10 mM pH 8.8. Whole-cell extracts and culture supernatants were then analyzed by Western blotting using polyclonal rabbit antibodies against EspA, EspB, EspD and Tir recombinant proteins as described by Vilte et al [46]. Primary antibodies were detected with horseradish peroxidase-conjugated goat anti-rabbit IgG (Sigma Chemical Co., St. Louis, MO) diluted 1∶10,000. The immunoblots were revealed with 4-Cl-1-naphthol (Pierce, Rockford, IL).

### EPEC-Secreted Proteins Associated to HEp-2 Cells and Bacterial Viability

HEp-2 monolayers were inoculated with bacteria pretreated with CoilA or CoilB peptides as described for fluorescent actin staining. After infection, the supernatant was removed from the plates and serial dilutions were cultured overnight at 37°C on LB agar plates to determine the viability of bacteria. In turn, the monolayers were resuspended in 40 µl of lysis buffer (1% Triton X-100) and protease inhibitor mix (Complete™ protease inhibitors, Roche). This material was subjected to Western blotting as mentioned above.

### Immunogold Labeling and Electronic Microscopy

EPEC E2348/69 strain was grown in LB overnight at 37°C without shaking and diluted 1∶100 into DMEM with 0.56 mg/ml of either CoilA or CoilB, in 24-well plates. The plates were incubated for 4 h under a 5% CO_2_ atmosphere. A 50 µl droplet of bacterial culture was applied to nickel EM grids (mesh 200) coated with 0.25% Formvar resin (Structure Probe, Inc., SPI supplies, PA, USA) for 2 min. Cells were immediately fixed face down for 15 min with 0.1% buffered glutaraldehyde in PBS (pH 7.3). The grids were washed by transfer across 6 drops of PBS and blocked in PBS containing 0.2% bovine serum albumin for 30 min at room temperature. The bacteria were then incubated on drops of 1∶100 polyclonal rabbit antibodies to EspA [46] in blocking buffer overnight at 4°C. Afterwards, the grids were washed by transfer across 6 drops of PBS and incubated with 1∶50 colloidal gold-conjugated goat affinity purified antibody to rabbit IgG (gold particle diameter, 5 nm) (Sigma Chemical Co., USA) for 2 h at room temperature. Grids were then washed with PBS, stained with 2% uranile acetate and examined by transmission electron microscopy (TEM) using a Jeol 1200 EXII equipment operating at 85 kV. Fifty EspA filaments were identified and the size was measured the length is given as an average with a standard deviation.

### Statistics

The values are expressed as mean ± standard error (mean ± SE). Statistical analysis was carried out using the Student's *t*-test, with a 95% confidence limit; a probability value of *P*<0.05 was considered significant.
